# Endoscopic treatment of Crohn-related strictures with a self-expandable stent compared with balloon dilation: a prospective, randomised, controlled study

**DOI:** 10.1136/bmjgast-2021-000612

**Published:** 2021-03-14

**Authors:** Per Hedenström, Per-Ove Stotzer

**Affiliations:** 1 Intitute of Medicine, Department of Molecular and Clinical Medicine, Gothenburg University, Gothenburg, Sweden; 2 Division of Gastroenterology, Department of Medicine, Sahlgrenska University Hospital, Gothenburg, Sweden

**Keywords:** fibrosis, endoscopy, stents, crohn's disease

## Abstract

**Objective:**

Fibrotic strictures in the gastrointestinal tract are frequent in Crohn’s disease. Endoscopic dilation is a standard treatment. However, recurrence is common after dilation and there are complications such as bleeding or perforation. Endoscopic treatment using self-expandable metal stents has shown diverging results. The aim of this study was to evaluate the outcome of endoscopic treatment with a self-expandable stent in ileocecal Crohn’s disease.

**Design/method:**

Patients with Crohn’s disease and a symptomatic ileocecal stricture were eligible for prospective, consecutive inclusion in a single-centre setting. Patients were randomised to treatment with either 18 mm balloon dilatation (Group_DIL_) or stenting (Group_STENT_) using a 20 mm diameter, partially covered Hanarostent NCN. Patients were followed for a minimum of 24 months postendoscopy. Outcomes were technical success, adverse events and clinical success (defined as no need for repeated interventions).

**Results:**

Thirteen patients (Group_DIL_ n=6; Group_STENT_=7) were included with twelve patients (Group_DIL_ n=5; Group_STENT_=7) being eligible for complete follow-up. Technical success was achieved in all cases. Adverse events were border-line significantly more common in the Group_STENT_: 4/7 (57%) (pain: n=3; pain and rectal bleeding: n=1) compared with the Group_DIL_: 0/5 (0%), p=0.08, which resulted in preterm termination of the study. The clinical success rate was Group_STENT_: 6/7 (86%) vs Group_DIL_: 1/5 (20%), p=0.07.

**Conclusion:**

Patients with strictures related to Crohn’s disease may benefit from treatment with self-expandable metal stents rather than dilatation. However, there seems to be an increased risk for patient pain after stenting, which has to be considered and handled.

**Trail registration number:**

The study was registered at Clinical Trials (NCT04718493).

**Summary box**

**What is already known about this subject?**

- Patients suffering from Crohn’s disease are under risk for developing fibrotic strictures, especially in the ileocecal region, which may cause severe symptoms and nutritional problems. Problematically, such strictures respond poorly to medical therapy and often the effect of endoscopic balloon dilatation is transitory. Therefore, patients may need surgical therapy, which is not without risk.

**What are the new findings?**

- In the current, prospective study we investigated the utility of endoscopic therapy using a self-expandable metal stent in patients with high-grade fibrotic strictures due to Crohn’s disease. Endoscopic balloon dilatation was used as comparison in a 1:1 randomised, head-to-head design. We found that the technical and clinical success rate of endoscopic stenting was high. Even though no severe adverse events in the form of perforation or major bleeding was recorded, stenting was associated with the risk for severe patient pain after the procedure. Therefore, the study was terminated preterm.

**How might it impact on clinical practice in the foreseeable future?**

- In patients suffering from Crohn’s disease, endoscopic therapy of fibrotic strictures using self-expandable metal stents might be considered by clinicians since the technique seems feasible and effective at long-term follow-up. However, the risk for patient pain poststenting appears high compared with endoscopic balloon dilation only. That risk has to be taken into account and handled if endoscopic stenting should be considered and justified.

## Background

One of the features of Crohn’s disease is fibrotic strictures in the gastrointestinal tract and the estimated incidence of strictures affecting the ileocecal region is 50%.^1^ In many cases, these strictures are resistant to medical treatment and the recurrence rate after surgical treatment is high.^2^

Endoscopic treatment using a hydrostatic balloon dilation is a well-established treatment option. The technical success rate of endoscopic balloon dilatation is high and major adverse events occur in 2 (0%–18)%.^3^ However, recurrences have been reported in up to 60% at 5 years after dilation.^4^ Importantly, the recurrence of strictures after balloon dilation implies that a new intervention, or even a surgical resection, will be needed.

Endoscopic stenting has been used as an alternative to balloon dilation but data are limited and show inconsistent results.^5–7^ Furthermore, in the available literature the protocols vary greatly concerning the types of stents used, the duration of stenting and the time for follow-up. The technical success rate of stenting has been reported more than 90% and the clinical success rate varies between 60% and 86%.^5 6^ Adverse events are frequent, especially a high stent migration rate.^5 7^ In one study, however, no migration was reported.^6^ Importantly, the migration rate is highly dependent on the type of stent used. Fully covered stents have a high rate of migration^8 9^ but a lower rate of ingrowth in the bowel wall. Large diameter stents are less prone to migrate than smaller diameter.^10–12^ Other reported adverse events are abscess, perforation and impaction.^13 14^

The aim of this study was to compare the outcome of endoscopic treatment using a self-expandable colonic stent with endoscopic balloon dilation only in patients with ileocecal Crohn’s disease.

## Methods

### Study subjects and setting

**Figure 1:**
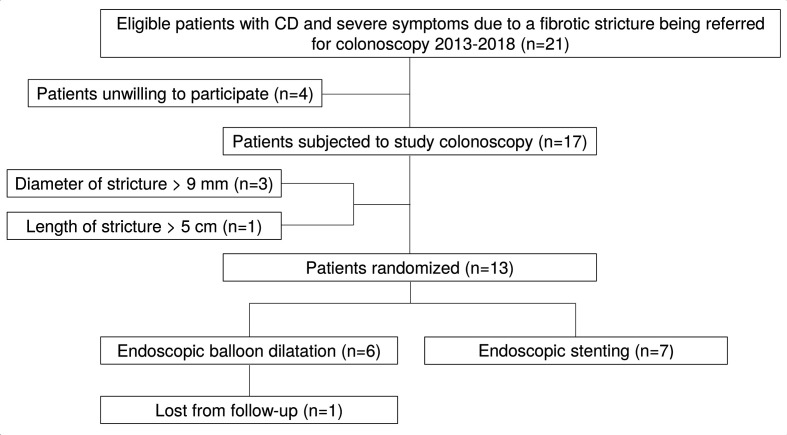
A flow chart of the study enrolment process. CD, Crohn’s disease.

This was a single-centre, prospective study performed at the endoscopy unit in Sahlgrenska University Hospital. Subjects aged >20 years of age diagnosed with Crohn’s disease and a symptomatic stricture in an ileocolic anastomosis or the ileocecal valve were eligible for consecutive study inclusion during the time frame 2013–2018 (figure 1). The stricture was to be of inflammatory origin, that is caused by long-standing Crohn inflammation. Only strictures with a lumen diameter of 9 mm or less were regarded as significant and thus included in the study. Strictures longer than 5 cm were not included. Other exclusion criteria were the suspicion of a malignant stricture, a pure postoperative anastomotic stricture not induced by Crohn’s inflammation, planned surgical therapy of the stricture or increased risk of bleeding.

Information and data on the baseline parameters, the diagnosis, the treatment and the course of disease in each patient, including imaging and laboratory tests, were collected from the medical files.

The steering group of the current study was the principal investigator (P-OS) together with the coworker (PH). The group was responsible for the major evaluations and decisions on the study such as the evaluations of any adverse events.

All patients gave written informed consent according to the Declaration of Helsinki.

### The endoscopic procedure

Patient consent was obtained prior to the colonoscopy but randomisation was done during the colonoscopy after assessment of the stricture. Colonoscopy was performed with a medium length colonoscope and a standard diameter working channel. First the ileocolic stricture was identified. Then, the stenosis was evaluated. If the stricture was 9 mm or less in diameter and not longer than 5 cm, the patient was randomised at a 1:1 ratio to endoscopic treatment with stenting (Group_STENT_) or to endoscopic treatment with balloon dilation only (Group_DIL_) by opening a sealed envelope. If the stricture was measured as wider than 9 mm or longer than 5 cm, the patient was excluded from study and these patients were not randomised. As by study subject #7, the study protocol of the stent treatment arm was modified from stenting only to balloon dilation followed by stenting in one procedure.

### The dilatation procedure

All procedures were made under fluoroscopic guidance with wire-guided technique. After inserting the guide wire through the stenosis, the position of the wire and the length of the stricture was controlled by injection of contrast. When satisfying position, the balloon (CRE, Boston Scientific, Marlborough, Massachusetts USA), was inserted over the wire and positioned. Then, the balloon was inflated to a diameter of 18 mm for 2 min. The effect of the dilation was controlled by passing the stricture with the colonoscope.

### The stenting procedure

All procedures were made under fluoroscopic guidance with wire-guided through the scope technique. For stenting a partially covered 20 mm diameter Hanarostent NCN (M.I. Tech, Korea) for Crohn’s disease was used. The stent has a flare in both ends to prevent migration. It also has two lassos to enable repositioning and removal of the stent.

After inserting the guide wire through the stenosis, the position of the wire and length of the stricture was controlled by injection of contrast. When satisfying position, the stent was inserted over the wire, positioned and released under fluoroscopic guidance.

After the modification of the study protocol, as by study subject #7, stenting was proceeded by dilation to 18 mm. To guarantee easy removal and to prevent ingrowth, the stent was removed after 7–10 days at a repeated colonoscopy.

### Follow-up

All study subjects were followed every 6 months for 2 years after the endoscopic procedure regarding general well-being, blood sample analysis, and if needed repeated imaging. Two years after the inclusion of the last patient, a review was performed of the medical files of all study subjects concerning any further interventions performed, that is, endoscopic or surgical treatment of Crohn-related strictures. Given the long time frame for inclusion of patients, some study participants could be monitored for longer than 24 months.

### Study outcomes

The primary study outcome was the technical success rate of stenting and balloon dilatation including the adverse event rate of each procedure.

Regarding balloon dilatation, technical success was defined as the uneventful introduction of the balloon catheter through the stricture, the completion of the 18 mm dilatation procedure as intended, and finally the passage of the colonoscope through the stricture. Regarding stenting, technical success was defined as the uneventful positioning and release of the stent at the intended location as measured by fluoroscopy.

An adverse event was defined as the development of symptoms likely related to the endoscopic procedure such as pain requiring treatment with analgesics, the occurrence of infection requiring treatment with antibiotics, or the occurrence of gastrointestinal bleeding. Any symptom, such as severe pain, requiring hospitalisation was regarded as a serious adverse event (SAE).

The secondary study outcome was the clinical success rate of stenting and balloon dilatation defined as a favourable clinical course without the need for any repeated endoscopic or surgical intervention.

In patients with a non-favourable clinical follow-up, the decision on reintervention or surgery was based on the combination of patient symptoms, findings at MRI, and, if performed, the results of colonoscopy. Patient symptoms alone were not regarded sufficient in this matter. The final decision on how to manage each patient was taken on an individual basis. The time point for any repeated intervention was recorded.

### Statistics

Descriptive, continuous data were described as mean and range, while descriptive, categorical data were described as frequencies.

Fisher’s exact test was used in the analysis of the outcome parameters comparing Group_STENT_ with Group_DIL_.

A sample size calculation was performed (statistical power: 80 %, alpha error: 0.05) to detect a 35%-difference in the clinical success rate of Group_STENT_ and Group_DIL_ (non-paired proportions, two sided). A value of n=28 required study cases was returned.

A two-tailed p<0.05 was regarded as statistically significant for all analyses.

All the statistical calculations and tests were performed using IBM SPSS Statistics V.25.0.

## Results

Thirteen patients (females: n=5; males n=8) were included in the study and twelve of these patients were eligible for analysis, (table 1). One patient (#1) was lost from follow-up and were not available in the analysis of adverse events and clinical success (figure 1). The mean age of the 12 patients was 56 years (range: 40–74) and 33% (4/12) were women. All patients had Crohn’s disease localised in the ileocecal region. The mean duration of the disease was 24 years. All but one subject had been subjected to a previous ileocecal resection.

**Table 1:** Baseline characteristics

Patient #	Age	Sex	Duration of disease (years)	Active smoking* (yes/no)	Therapy azathioprin* (yes/no)	Therapy anti-TNF alpha* (yes/no)
1	55	F	31	Yes	No	No
2	69	F	30	Yes	Yes	Yes
3	60	F	30	No	Yes	No
4	41	M	20	No	Yes	No
5	57	M	40	No	Yes	No
6	59	F	12	Yes	No	Yes
7	60	M	29	Yes	No	Yes
8	40	M	20	No	Yes	No
9	74	M	20	No	No	Yes
10	58	M	15	Yes	No	No
11	50	M	25	No	Yes	No
12	59	M	24	No	No	No
13	52	F	25	No	No	No

*Smoking status and therapy status are presented as by the date of the endoscopic procedure.

TNF, tumour necrosis factor.

Five patients were treated with balloon dilation only (four men and one woman) and seven patients were treated with stenting (four men and three women). Three patients treated with stenting had stenting only and the other four patients had stenting preceded by balloon dilatation.

The mean follow-up time after the study procedures was 69 months (range: 28–91).

### The primary outcome

All 13 procedures were technically successful, technical success rate 100%, including the removal of all stents after 7–10 days table 2.

**Table 2:** Procedure characteristics and study outcome

Patient #	Group	Intervention	Technical success	Adverse event	Follow-up (months)	Disease status postendoscopy*	Clinical success	Reintervention
1	Group_DIL_	Dilatation	Yes	Lost	Lost	Lost	Lost	Lost
2	Group_DIL_	Dilatation	Yes	None	92	Active	No	Surgical resection
3	Group_STENT_	Stent	Yes	Pain +Bleeding	91	Remission	Yes	None
4	Group_STENT_	Stent	Yes	Pain	91	Remission	No	Surgical resection
5	Group_DIL_	Dilatation	Yes	None	87	Remission	No	Re-dilatation
6	Group_STENT_	Stent	Yes	Pain	77	Remission	Yes	None
7	Group_DIL_	Dilatation	Yes	None	76	Active	No	Surgical resection
8	Group_STENT_	Dilatation +Stent	Yes	None	76	Active	Yes	None
9	Group_STENT_	Dilatation +Stent	Yes	None	65	Active	Yes	None
10	Group_DIL_	Dilation	Yes	None	59	Remission	No	Redilatation
11	Group_STENT_	Dilatation +Stent	Yes	None	57	Remission	Yes	None
12	Group_DIL_	Dilatation	Yes	None	33	Remission	Yes	None
13	Group_STENT_	Dilatation +Stent	Yes	Pain	28	Remission	Yes	None

*The disease status in the strictured region after endoscopy was monitored via imaging, and laboratory tests and based on the findings it was assessed as active (with signs of significant ongoing inflammation) or in remission (without signs of significant ongoing inflammation).

No adverse events were recorded in the group of patients subjected to balloon dilation only (Group_DIL_ adverse event rate: 0/5 (0%)). In the group of patients treated with stenting, an adverse event was recorded in four patients (Group_STENT_ adverse event rate: 4/7 (57%)). The difference in the adverse event rate comparing the two groups was borderline significant, p=0.08.

Among the four patients of the stent group with adverse events, patient number #3 had pain after the treatment and required treatment with oral analgesics. The very same patient also required overnight stay in the hospital 8 days after stent extraction due to a rectal bleeding.

However, no specific treatment was necessary. Patient number #4 and #6 also experienced pain after the procedure and during the stent period. Both required treatment with oral analgesics but no stay in the hospital. Finally, patient number #13 (stenting preceded by balloon dilatation) had to be hospitalised overnight due to severe pain after stenting. Hence, the rate of SAE in the Group_STENT_ was 2/7, 29%.

Then, it was decided that the study should be terminated. Nevertheless, in the four patients who experienced pain after stenting, as described above, treatment with analgesics was sufficient and all four stents could be left in place until planned removal of the stents (table 2).

### The secondary outcome

In the Group_DIL_, the clinical success rate was 1/5 (20%). Four patients required a new intervention; two patients had a repeated dilation and two patients required surgical resection (table 2). In the Group_STENT_, the clinical success rate was 6/7 (86 %). The only patient with a non-favourable clinical course had a new surgical resection of the ileocecal stenosis. Comparing the two groups, the secondary outcome was borderline significant, p=0.07, in favour of stenting (table 2).

## Discussion

To the best of our knowledge, this is the first prospective, randomised, controlled study comparing balloon dilatation with stenting in patients with a fibrotic stricture related to Crohn’s disease. The technical success rate of either technique was high. Six out of seven patients treated with a self-expandable stent achieved clinical success and did not need any further invasive procedure during the relatively long follow-up time. As comparison, four out of five patients treated with balloon dilatation only, required further endoscopic or surgical treatment. However, we recorded an equally strong tendency to a higher adverse event rate in the stenting group, which might limit the use of stenting as a primary treatment option in patients with strictures induced by Crohn’s disease.

A high proportion of subjects in the stent group had adverse events. Two patients had stent related pain, which required treatment with analgesics only. The other two patients had somewhat more intense pain and had to be monitored in hospital overnight including intravenous analgesics. One of these four subjects also had a late rectal bleeding after stent extraction but there was no need for interventional treatment of the bleeding, which ceased spontaneously. Despite the adverse events recorded, no case had serious complications in the form of severe bleeding, perforation or infection. One possible explanation for the adverse events recorded is that we used another type of stent compared with most other studies. Possibly due to a higher dilation force in the stricture, the design of the stent used in the current study better protects from migration but to the cost of patient pain. None of the patients in the study had stent migration, which is a relatively common phenomenon according to the existing literature.^13^ The duration of the stent period was limited to 7–10 days, mainly to prevent ingrowth. Hypothetically, this relatively short duration might prevent migration in some cases. Moreover, we applied strict criteria for inclusion in the study. Only patients with narrow, symptomatic strictures were included, which may have contributed to the absence of stent migration. The latter hypothesis goes is in line with a previous report using a similar setting.^6^

After careful consideration, we decided to terminate the study preterm due to the recorded events of patient pain. Nevertheless, especially given the probable, long-term positive clinical outcome in patients treated with stenting, the use of self-expandable metal stent might be justifiable in a selected group of patients with severe symptoms. Moreover, no severe complication and no permanent morbidity was recorded in any of the patients. Accurate information to patients about the risk for pain and prophylactic treatment with analgesics during the week after stenting, might reduce the problem with pain poststenting.

The decision to terminate the study was a delicate one but at the time point for study termination, we did not have any favourable follow-up data that could clearly motivate the proceeding of the study from a risk–benefit point of view. If we would have had more solid data on the positive clinical effects of stenting, that could have been a reason to continue with the study since that could have counterbalanced the risk for poststenting pain. Not to tell patients about the risk for pain after stenting would have been questionable from an ethical point of view.

Another decision that motivates discussion, was the decision to modify the study protocol after patient #6, that is to perform dilatation before stenting. Obviously, such a modification might introduce bias and complicate the interpretation of the results. However, we assessed this risk for bias as comparatively low and the modification was warranted by the recorded poststenting pain recorded in all the first three patients randomised to stenting.

In our study, and as compared with previous publications, we recorded a relatively high rate of reinterventions in the group of patients subjected to balloon dilatation only.^3^ One explanation of this finding could be that the available follow-up time after dilation was considerably longer in many of the patients included in our study as compared with previous ones.^3^ Since recurrence tend to increase over time the suggested explanation is reasonably likely.^3^ Even though all patients were followed for a minimum of 24 months, it should be taken into account that the course of disease could be followed in some of the study patients for a longer time than that. This circumstance should be remembered when interpreting the presented results.

Interestingly, the need for reinterventions in the stenting group was low with only one patient being referred for surgery of the stented stenosis. Hence, the effect of stenting seems to be durable over time, which would be a great advantage in this specific group of patients with Crohn’s disease. Obviously, this finding needs to be confirmed by others.

An obvious drawback of stenting is the need for a repeated colonoscopy within 2 weeks for the removal of the stent. This circumstance might have a negative impact on the implementation of stenting as the primary approach. Indeed, in the current study four eligible patients denied to participate because of the risk for a repeated colonoscopy (if randomised to the stent group). The removal procedure was also the probable cause of bleeding in one case as described above.

One strength of the present work is that, the study was conducted in a prospective setting applying a careful study protocol and strict inclusion criteria. Moreover, one single endoscopist performed all the study endoscopy procedures. Another advantage was the long follow-up period, which is of true interest to exclude temporary effects and benefits of whatever treatment applied.

Unfortunately, the current study had to be interrupted preterm due to the recorded four patients with pain after stenting. Originally, our aim was to include at least 28 patients in the study, but in the light of the recorded adverse events continued inclusions would have been ethically questionable. Another weakness of the study was that one of the patients was lost from the follow-up. A final weakness of the current work is the single-centre design, which limits the pace of patient inclusion and the external validity of the results. The intention was to expand the study to a multi-centric one but at that time we had already decided to terminate the study.

In conclusion, patients with fibrotic strictures related to Crohn’s disease might benefit from endoscopic treatment with self-expandable stents. However, there seems to be a significant risk for patient pain poststenting compared with endoscopic balloon dilation only. That risk has to be taken into account and handled, by for example mandatory analgesics 1 week after stenting, if endoscopic stenting should be considered and justified. The use of an alternative stent or a modification of the stenting procedure might decrease the risk for patient pain.

## Data Availability

Data are available on reasonable request.
